# Up-regulation of BRCA1-associated RING Domain 1 Promotes Hepatocellular Carcinoma Progression by Targeting Akt Signaling

**DOI:** 10.1038/s41598-017-07962-7

**Published:** 2017-08-09

**Authors:** Yan Liao, Shengguang Yuan, Xinhuang Chen, Pengpeng Zhu, Jun Li, Liling Qin, Weijia Liao

**Affiliations:** 1 Laboratory of Hepatobiliary and Pancreatic Surgery, Affiliated Hospital of Guilin Medical University, Guilin, Guangxi P.R. China; 2 Disease Prevention and Control Center of Guilin, Guilin, Guangxi P.R. China; 3 Department of Hepatobiliary and Pancreatic Surgery, Affiliated Hospital of Guilin Medical University, Guilin, Guangxi P.R. China

## Abstract

The present study was designed to investigate the potential clinical, pathological, prognostic value, role and mechanism of BRCA1-associated RING Domain 1 (BARD1) in Hepatocellular carcinoma (HCC). Quantitative real-time PCR and immunohistochemistry were performed to evaluate the expression of BARD1 mRNA and protein. The expression of BARD1 in the HCC tissue samples was markedly higher than that in the adjacent noncancerous liver tissues. Elevated BARD1 expression was positively correlated with tumor-node-metastasis stage, Barcelona-Clinic Liver Cancer stage, hepatitis B surface antigen, large tumor size, serum alpha-fetoprotein levels, and serum aspartate aminotransferase levels. Univariate and multivariate analyses revealed the BARD1 was an independent predictor for decreased progression-free survival and overall survival in HCC. *In vitro* experiments demonstrated that knocking down BARD1 significantly inhibited the proliferation, invasion and migration of HCC cells. Moreover, silencing BARD1 inhibit the signaling pathway via decreased the levels of Akt, mTOR, and MMP-9 and inhibited the phosphorylation of Akt (Ser473) and mTOR (Ser2248). Collectively, our findings suggest that BARD1 may be a novel diagnostic and prognostic biomarker of HCC, and up-regulation of BARD1 can contribute to HCC progression by targeting Akt signaling.

## Introduction

Hepatocellular carcinoma (HCC) is ranked the fifth most common malignant cancer worldwide, and it contributes to more than 90% of all liver cancers^1, 2^. Although current therapies, including surgical resection or liver transplantation, have improved the quality life of HCC patients to some extent, prognosis is still dismal for most HCC patients due to intrahepatic recurrence or tumor metastases^3, 4^. Growing evidence supports the notion that the various accumulated genetic alterations that occur in hepatocytes and the changes in the subsequent signaling pathways may regulate the initiation and progression of HCC^5, 6^. The exploration of possible molecular characteristics that lead to tumor proliferation, migration and metastasis is necessary to identify novel therapeutic targets for HCC patients.

In the previous genome-wide microarray analysis, we found that the BRCA1-associated RING Domain 1 (BARD1) was significantly up-regulated in HCC tissues. Previous studies also have found that BARD1 expression was highly up-regulated in breast and non-small cell lung cancer^7, 8^. BARD1 was identified as a nuclear protein that interacts with BRCA1 both during the S phase of cell division and after DNA damage^9–11^. BARD1 and BRCA1 form a stable heterodimer that has been implicated in DNA repair foci, transcription regulation, centrosome duplication, ubiquitination, and mitotic spindle assembly, which are crucial functions for the maintenance of organism genome integrity^12–15^.

Mutations in BARD1 gene may lack the RING domain that interacts with BRCA1, thereby affecting the sub-cellular localization and retention of BRCA1 and compromising tumor suppressor activity; these mechanisms may contribute to carcinogenesis^16–18^. A significant up-regulation of BARD1 expression was observed in the cytoplasm of most cancer cells, but BARD1 expression was down-regulated in the surrounding healthy tissue^19^. It also has been reported that BARD1 mutations are associated with an increased risk of inherited and spontaneous breast and ovarian cancers^20–22^.

In the present work, we aimed to fully characterize the clinical of BARD1 in HCC. Our findings show a significant increase in BARD1 expression in human HCC specimens compared to adjacent non-cancerous tissues (ANLT). Moreover, elevated BARD1 expression predicts poor progression-free survival (PFS) and overall survival (OS) of HCC patients.

## Materials and Methods

### Tissue samplesh

The current study was approved by the research ethics committee of Affiliated Hospital of Guilin Medical University (Guangxi, China). Written informed consent was obtained from all recruited HCC patients, according to our university guidelines. All methods were carried out in accordance with Affiliated Hospital of Guilin Medical University guidelines and regulations. All experimental protocols were approved by Affiliated Hospital of Guilin Medical University. Informed consent was obtained from all patients for use of the tissues in experimental procedures. Tissue specimens came from 209 HCC patients. Samples of matched tumor-adjacent normal tissues were obtained between March 2001 and December 2007 at the Affiliated Hospital of Guilin Medical University (Guangxi, China). The patients underwent routine clinical, pathological, blood, ultrasonography (US), computed tomography (CT) scan, and magnetic resonance imaging (MRI) investigations. The clinical diagnosis of HCC was based on histological analysis. Staging of HCC was determined according to the tumor-node-metastasis (TNM) classification system and the Barcelona Clinic Liver Cancer (BCLC) staging protocol. The full electronic medical records of all HCC patients are shown in Table 1. The 209 paired samples of HCC tissues and their matched ANLT, were frozen and stored at −80 °C for the qRT-PCR assay. Additionally, a total of 70 paired samples of HCC tissues and tumor-adjacent normal tissues were quickly processed into 10% formalin and subsequently embedded in paraffin wax for immunohistochemical analysis. The inclusion criteria of this study were as follows: 1) HCC samples obtained by radical resection and confirmed by pathology and 2) complete patient background information and follow-up records, including periodic liver function tests, AFP values, imaging information, and the recurrence time and subsequent treatment. The exclusion criteria were as follows: 1) patients without HCC; 2) patients who died perioperatively; 3) incomplete clinical data; 4) patients who suffered from infectious disease, immune system disorders, hematological disease or used hematological drugs within one month; 5) patients who lost contact in the follow-up period; and 6) patients positive for HIV. The study was approved by the Ethics Committee of Affiliated Hospital of Guilin Medical University and complied with the Ethical Guidelines of the Declaration of Helsinki. Informed consent from patients or family members was obtained prior to the investigation.

**Table 1:** Correlation between the clinicopathologic variables and BARD1 in HCC.

Clinical character	Clinical variable	No. of patients	BARD1 mRNA		χ^2^	*P* value
			low n (%)	high n (%)		
Gender	Female	33	7 (21.2)	26 (78.8)	1.485	0.223
	Male	176	56 (31.8)	120 (68.2)		
Age (years)	≤55	141	39 (27.7)	102 (72.3)	1.270	0.260
	>55	68	24 (35.3)	44 (64.7)		
Family history	No	176	51 (29.0)	125 (71.0)	0.720	0.396
	Yes	33	12 (36.4)	21 (63.6)		
HBsAg	Negative	39	19 (48.7)	20 (51.3)	7.856	0.005
	Positive	170	44 (25.9)	126 (74.1)		
Median size (range, cm)	≤5	73	31 (42.5)	42 (57.5)	8.089	0.004
	>5	136	32 (23.5)	104 (76.5)		
Cirrhosis	No	20	3 (15.0)	17 (85.0)	2.409	0.121
	Yes	189	60 (31.7)	129 (68.3)		
Tumor number	Single	141	46 (32.6)	95 (67.4)	1.266	0.260
	Multiple	68	17 (25.0)	51 (75.0)		
Wine-drinking	No	103	32 (31.1)	71 (68.9)	0.082	0.774
	Yes	106	31 (29.2)	75 (70.8)		
TNM stage	I–II	91	39 (42.9)	52 (57.1)	12.372	<0.001
	III–IV	118	24 (20.3)	94 (79.7)		
Metastasis	No	178	55 (30.9)	123 (69.1)	0.325	0.569
	Yes	31	8 (25.8)	23 (74.2)		
BCLC stage	0-A	96	38 (39.6)	58 (60.4)	7.514	0.006
	B–C	113	25 (22.1)	88 (77.9)		
AFP (ng/mL)	≤100	84	34 (40.5)	50 (59.5)	7.121	0.008
	>100	125	29 (23.2)	96 (76.8)		
AST (U/L)	≤40	97	39 (40.2)	58 (59.8)	8.704	0.003
	>40	112	24 (21.4)	88 (78.6)		
Recurrence	No	135	39 (28.9)	96 (71.1)	0.285	0.593
	Yes	74	24 (32.4)	50 (67.6)		

HBsAg, hepatitis B surface antigen; TNM, tumor-node-metastasis; Metastasis, distant metastasis or lymph node metastasis; BCLC, Barcelona-Clinic Liver Cancer; AFP, alpha-fetoprotein; AST, aspartate aminotransferase.

All HCC patients had been followed up by monitoring their serum alpha-fetoprotein (AFP) level, abdominal ultrasonography, and chest radiography every 6 months for the first 2 years after the operation and every 3–6 months in subsequent years. Diagnosis of tumor recurrence was confirmed via ultrasonography, dynamic CT scan, MRI, and AFP levels. The median time of follow-up was 36.0 months (median, 21.0 months; range, 1.0 to 84.0 months) for survivors. PFS was defined from the date of resection to the date of recurrence, metastasis, death or the latest follow-up visit. OS time was defined as the interval between the operation and death or the latest follow-up visit.

### Reverse transcription-PCR and quantitative real-time PCR (qRT-PCR)

Total RNA from HCC tissues, matched tumor-adjacent normal tissues and HCC cell lines were isolated with the Trizol reagent (Invitrogen, Carlsbad, CA, USA) in accordance with the manufacturer protocol. RNA samples were reverse transcribed into first-strand cDNA by the Prime Script RT Reagent Kit (TaKaRa, Otsu, Japan) according to manufacturer instructions. For quantification of the BARD1 transcript relative to the β-actin gene, we used the primer sequences for BARD1 (forward: 5′-GCTTCTATTAAGGGCGACAT-3′; reverse: 5′-GTTCACCAATGCCTTATGCT-3′); the length of the amplified fragment was 162 bp. The levels of BARD1 genes were normalized to expression levels of the β-actin gene (forward: 5′-GACAGGATGCAGAAGGAGATTACT-3′; reverse: 5′-TGATCCACATCTGCTGGAAGGT-3′), which generated a 142 bp fragment. qRT-PCR was performed with the ABI 7500 system at the following cycle: 95 °C for 10 min, followed by 40 cycles of denaturation at 95 °C for 30 sec, annealing at 55 °C for 30 sec and extension at 72 °C for 30 sec, and fluorescence acquisition at 72 °C. Each PCR mixture (20 μl) included 10 μl SYBR Green PCR Master Mix (Applied Biosystems, Foster City, CA, USA), 0.2 μl of each primer, and 1 μl of template. Data analyses for the BARD1 gene expression was performed using the method previously described^23^.

### Immunohistochemistry (IHC) analysis

IHC analysis was used to examine BARD1 protein expression in 70 human HCC tissues. Briefly, paraffin tissue sections were deparaffinized with xylene and rehydrated with a graded series of ethanol and distilled water. Antigen retrieval was performed with citrate antigenic retrieval buffer at pH 6.0 for 3 minutes in a pressure cooker. After blocking endogenous peroxidase activity with 3% H_2_O_2_ for 10 min, the samples were then treated with 10% goat serum at room temperature for 30 min to block nonspecific binding. The tissue sections were then incubated with rabbit anti-BARD1 antibodies (ab115477, Abcam Company, 1:200 dilution) overnight at 4 °C. The following day, after washing, the sections were incubated with peroxidase-conjugated goat anti-rabbit antibody at room temperature for 1 hour. Subsequently, the tissue sections yielded coloration with 3,3- diaminobenzidine tetrahydrochloride (DAB) and were further stained with haematoxylin. Normal rabbit serum instead of the primary antibody was used as a negative control, whereas human skeletal muscle, in which BARD1 is known to be expressed, was used as positive control in each experiment. BARD1 expression was assessed by two independent pathologists who were blinded to the histopathological features and patient data. The positively stained tumor cells were semi-quantitatively assessed, and each sample was graded as described previously^23^.

### RNA interference

Three siRNAs against BARD1 were designed and synthesized by GenePharma Con., Ltd (Shanghai). The sequences of the siRNAs were as follows: siRNA-1: 5′-GCUGCUCGCGUUGUACUAATT-3′ and 5′UUAGUACAACGCGAGCAGCTT-3′; siRNA-2: 5′-GCUCCAUAUUGCUUCUAUUTT-3′, and 5′-AAUAGAAGCAAUAUGGAGCTT-3′; siRNA-3: 5′-GCUAGCCACUGCUCAGUAATT-3′, and 5′-UUACUGAGCAGUGGCUAGCTT-3′. The negative control (siRNA-NC: 5′-GAGUUAAAGUCAAAGUGACTT-3′, 5′-GUCACUUUGACUUUAACUCTT-3′) was also synthesized.

### Cell culture and transfection

HCC cell lines SMMC7721 and Huh7 were purchased from institute of chemistry and cell biology (Shanghai, China). Both of the cell lines were cultured in Dulbecco’s modified Eagle’s medium (DMEM; Gibco, Grand Island, NY, USA) containing with 10% fetal bovine serum (FBS; Gibco) and maintained at 37 °C and 5% CO_2_ incubator. SiRNAs were transfected into target cells using Lipofectamine™ 3000 (Invitrogen) according to the manufacturer’s instructions at a 60%-70% confluence.

### Colony formation assay

The siRNA-transfected cells were seeded in six-well plates (600 cells/well) and cultured in DEME medium with 1% FBS for two weeks. Then the cells were washed three times with PBS, fixed with 4% paraformaldehyde for 30 min and stained with 0.1% crystal violet overnight. After extensive washing and air drying, plates were photographed and colonies were counted.

### Cell invasion and migration assay

Cell invasion assay was performed by using 24-well Transwells (8-μm pore size; BD Biosciences) that were coated with 10 ul Matrigel (Falcon 354480; BD Biosciences) as previously described^23^. The experimental procedures of cell migration assay were similar to the cell invasion assay, except that the 24-well Transwells were inserted with polycarbonate membrane filter (8 μm pore size; BD Biosciences) without Matrigel.

### Western Blotting

After transfection, the total cell protein was extracted by using lysis buffer (Beyotim Biotechnology). Protein samples were separated by 10% SDS-PAGE and transferred to polyvinylidene difluoride (PVDF) membranes (BioRad). The membranes were blocked with 5% skim milk for 1 hour and then incubated with primary antibody overnight at 4 °C. Primary antibodies used for monitoring protein expression were as follows: BARD1 antibody (ab69645, rabbit polyclonal antibody, 86 kDa, dilution 1:500, Abcam), Akt antibody (#4685, rabbit monoclonal antibody, 60 KDa, dilution 1:1000, Cell Signaling Technology), phospho-Akt (Ser473) antibody (#12694, rabbit monoclonal antibody, 60 KDa, dilution 1:1000, Cell Signaling Technology), mTOR antibody (#2972, rabbit polyclonal antibody, 289 KDa, dilution 1:1000, Cell Signaling Technology), phospho-mTOR (Ser2448) antibody (#2971, rabbit polyclonal antibody, 289 KDa, dilution 1:1000, Cell Signaling Technology), MMP-9 antibody (#13667, rabbit monoclonal antibody, 84 KDa, dilution 1:1000, Cell Signaling Technology), P53 antibody (#4667, rabbit monoclonal antibody, 53 KDa, dilution 1:1000, Cell Signaling Technology). β-actin (#12620, rabbit monoclonal antibody, 45 KDa, dilution 1:1000, Cell Signaling Technology) was used as internal positive control. After applying the affinity purified HRP-conjugated goat anti-rabbit secondary antibody, immunoreactive proteins were visualized by chemiluminescence and exposed on X-ray films (Fuji films).

### Statistical methods

Statistical analyses were performed with SPSS 18.0 (SPSS Inc, Chicago, IL). The relationships between BARD1 expression and clinicopathologic features were examined by the Pearson χ^2^ test or the Fisher exact test. Quantitative variables were assessed by Student’s t test. The survival rate was performed using the Kaplan-Meier method, and the difference in survival rates was compared using the log-rank test. A Cox proportional-hazard model was used to determine independent prognostic factors based on the variables affected on survival in univariate analyses. *P* < 0.05 was considered statistical significance.

## Results

### Elevated expression of BARD1 in hepatic cancer tissues at both mRNA and protein levels

**Figure 1:**
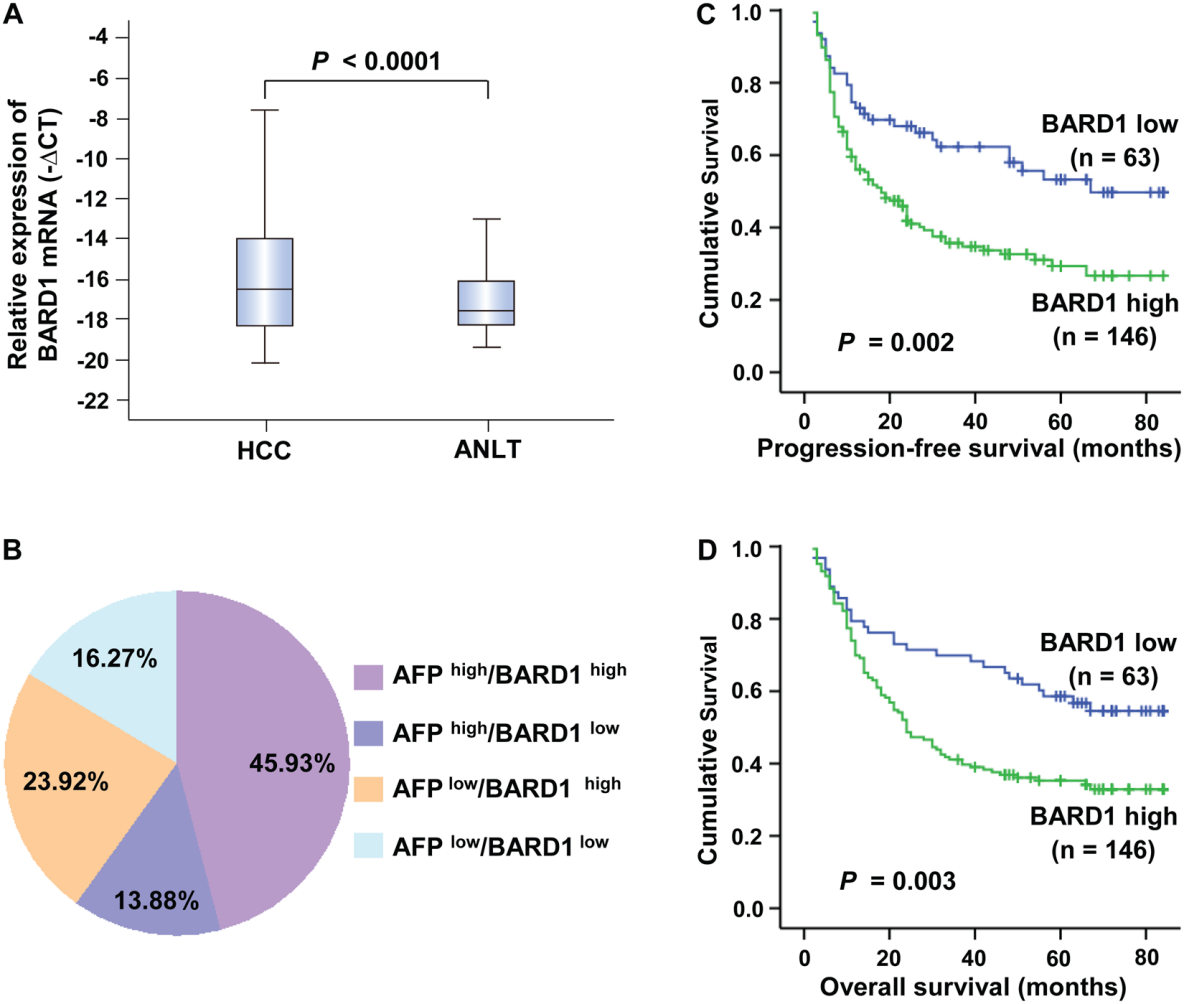
BARD1 mRNA up-regulation in HCC and the relationship with patient survival. (**A**) qRT-PCR analysis of BARD1 was carried out in 209 paired HCC and matched ANLT. For each sample, the relative expression level of BARD1 was normalized based on that of the internal reference β-actin, the line within each box represents the median −ΔCt value. (**B**) The distribution of both BARD1 mRNA expression and serum AFP levels in 209 HCC patients; the numbers in the pie indicate the percentages of BARD1 and/or AFP whose level is high or low in HCC specimens. Patients with high BARD1 expression profile had a poorer outcome for both PFS (**C**) and OS (**D**) among the HCC patients.

**Figure 2:**
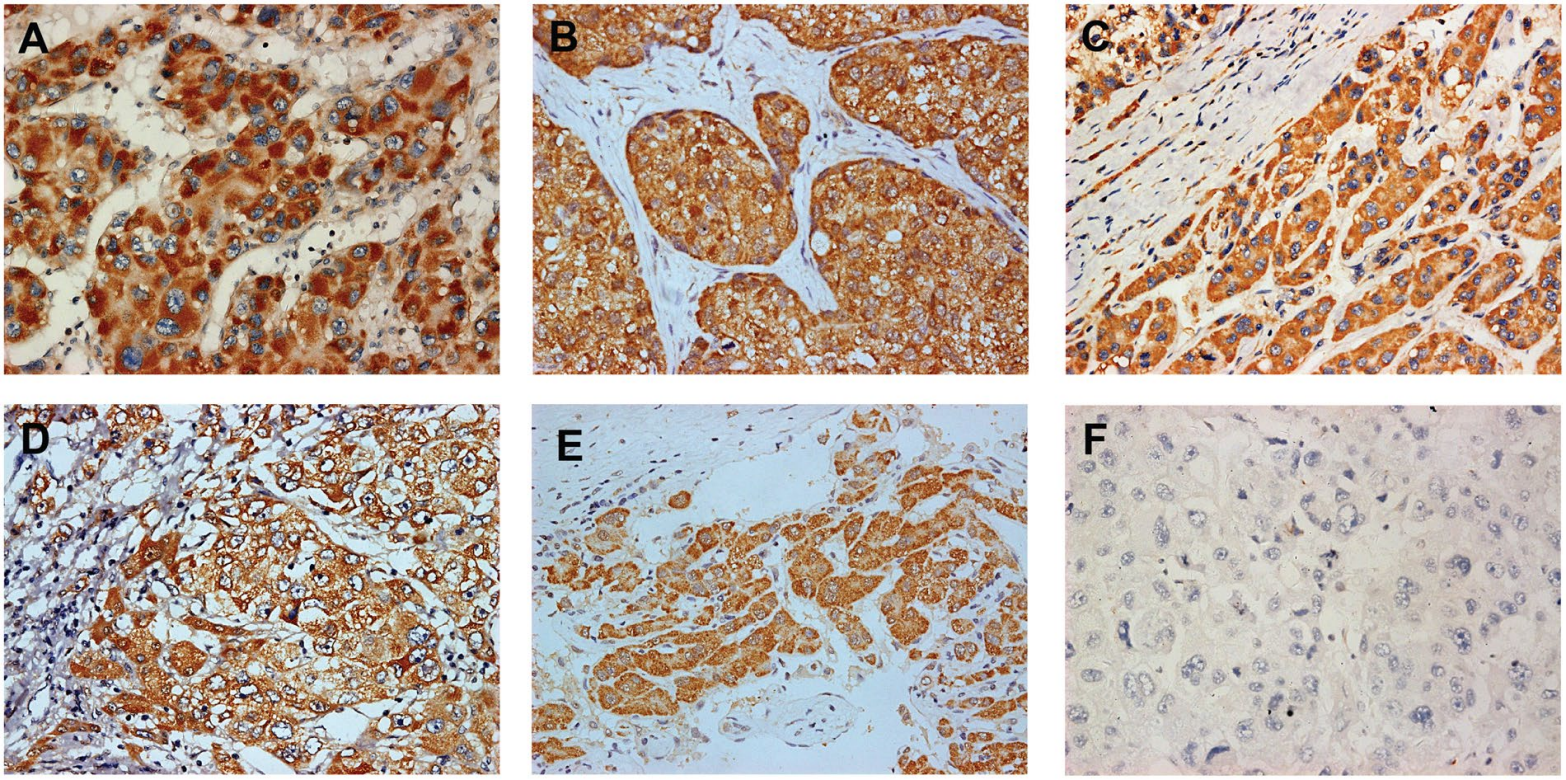
The protein level of BARD1 in HCC was determined by IHC. (**A–F**) Immunohistochemistry analysis for BARD1 level in 6 representative cases. BARD1 was stained in brown color, positively stained positive (**A–E**) and negative (**F**). The nuclei were counterstained with hematoxylin. Original magnification: ×200.

BARD1 expression was initially detected in HCC samples, adjacent normal liver tissues, and normal liver tissues from hepatic hemangiomas surrounding the liver tissues by qRT-PCR and IHC. We found that the BARD1 mRNA expression was significantly up-regulated in HCC tissues compared to the ANLT (Fig. 1A, *P* < 0.0001). The results of the IHC assay also verified these findings, six representative cases in which the positively stained positive or negative with anti-BARD1 are shown in Fig. 2. BARD1 protein expression was observed in 50 of the 70 HCC specimens (71.43%); however, only 20 (28.57%) ANLT had positive BARD1 staining, and the difference in BARD1 staining between the HCC and ANLT was statistically significant (*P* < 0.001).

### Value of combined BARD1 expression and AFP in HCC diagnosis

The diagnostic value of combined BARD1 expression and AFP estimation were then studied by the correlation analysis. We found that 45.93% (96/209) of the patients exhibited BARD1 ^high^ and AFP ^high^, 13.88% (29/209) of the individuals exhibited BARD1 ^low^ and AFP ^high^, and the subgroup with BARD1 ^high^ and AFP ^low^ included 23.92% (50/209) of the patients (Fig. 1B). These findings indicate that the combination of BARD1 and AFP significantly improved the diagnostic power in HCC compared to each marker alone. The simultaneous detection of AFP and BARD1 could increase the diagnostic sensitivity to 83.73%.

### Relationships between BARD1 and clinicopathological features in HCC

To evaluate the biological significance of BARD1, we assessed the association between BARD1 and the clinicopathological characteristics of HCC. As shown in Table 1, BARD1 mRNA expression level in HCC tissues was significantly associated with TNM stage (*P* < 0.001), BCLC stage (*P* = 0.006), hepatitis B surface antigen (HBsAg) status (*P* = 0.005), size of tumor (*P* = 0.004), serum AFP levels (*P* = 0.008), and serum aspartate aminotransferase (AST) levels (*P* = 0.003). However, no significant relationship was observed between the BARD1 expression level and the other clinical parameters, such as age, gender, family history, cirrhosis, tumor number, wine-drinking, metastasis, and recurrence (all *P* > 0.05, Table 1).

### High BARD1 expression is significantly associated with poor prognosis of HCC patients

Kaplan-Meier analysis and the log-rank test were used to assess the relationship between BARD1 mRNA expression and the PFS and OS of patients. Patients with high levels of BARD1 had a shorter PFS [34.86 months; 95% confidence interval (CI): 29.23–40.49] than those with low levels of BARD1 [53.01 months; 95% CI: 44.29–61.73] (*P* = 0.002, Fig. 1C). Similarly, the OS was significantly lower in patients with high levels of BARD1 (40.33 months; 95% CI: 34.98–45.69) than in those with low levels of BARD1 (57.31 months; 95% CI: 49.25–65.36) (*P* = 0.003, Fig. 1D).

In the univariate survival analysis, we detected correlations between different parameters and the survival rate of HCC patients. High tumor expression of BARD1 (*P* = 0.002), median tumor size >5 cm (*P* < 0.001), multiple tumor numbers (*P* < 0.001), TNM stage III–IV (*P* < 0.001), higher BCLC stage (*P* < 0.001), and increased serum AST levels (*P* < 0.001) were identified as parameters significantly associated with PFS of HCC patients (Table 2). Elevated tumor expression of BARD1 (*P* = 0.003), size of tumor >5 cm (*P* < 0.001), multiple tumor numbers (*P* < 0.001), TNM stage III–IV (*P* < 0.001), higher BCLC stage (*P* < 0.001), increased serum AST levels (*P* < 0.001), and recurrence (*P* < 0.001) were significant prognostic factors for OS in HCC patients (Table 3

**Table 2:** Univariate and multivariate analysis of progression-free survival.

Clinical character	Category	No.of patients	Univariate analysis			Multivariate analysis	
			Mean	95% CI	*P* value	HR (95% CI)	*P* value
BARD1 expression	Low	63	53.01	44.29–61.73	0.002	1.769 (1.184–2.644)	0.005
	High	146	34.86	29.23–40.49			
Gender	Female	33	42.42	31.42–53.42	0.344		
	Male	176	39.47	34.28–44.67			
Age (years)	≤55	141	39.80	33.92–45.69	0.754		
	>55	68	41.74	33.13–50.34			
Family history	No	176	38.86	33.60–44.12	0.170		
	Yes	33	48.85	36.88–60.83			
HBsAg	Negative	39	37.92	26.89–48.94	0.863		
	Positive	170	40.86	35.45–46.27			
Median size (range, cm)	≤5	73	62.47	55.22–69.72	<0.001	2.420 (1.468–3.991)	0.001
	>5	136	28.60	23.20–33.99			
Cirrhosis	No	20	37.32	21.97–52.66	0.687		
	Yes	189	40.77	35.65–45.89			
Tumor number	Single	141	46.61	40.64–52.58	<0.001	1.173 (0.727–1.894)	0.513
	Multiple	68	27.08	19.75–34.41			
Wine-drinking	No	103	45.19	37.84–52.54	0.125		
	Yes	106	36.44	30.14–42.75			
TNM stage	I–II	91	55.82	48.91–62.73	<0.001	1.698 (1.026–2.809)	0.039
	III–IV	118	28.54	22.62–34.47			
Metastasis	No	178	41.98	36.67–47.29	0.122		
	Yes	31	30.57	19.58–41.56			
BCLC stage	0-A	96	55.24	48.29–62.20	<0.001	1.156 (0.631–2.116)	0.639
	B–C	113	27.68	21.88–33.48			
AFP (ng/ml)	≤100	84	44.22	36.53–51.90	0.177		
	>100	125	37.97	31.73–44.20			
AST (U/L)	≤40	97	54.56	47.42–61.69	<0.001	1.664 (1.072–2.582)	0.023
	>40	112	28.51	22.79–34.23			

HR, hazard ratio; CI, confidence interval.

**Table 3:** Univariate and multivariate analysis of overall survival.

Clinical character	Category	No.of patients	Univariate analysis			Multivariate analysis	
			Mean	95% CI	*P* value	HR (95% CI)	*P* value
BARD1 expression	Low	63	57.31	49.25–65.36	0.003	1.655 (1.100–2.489)	0.016
	High	146	40.33	34.98–45.69			
Gender	Female	33	53.26	41.42–65.10	0.164		
	Male	176	43.97	39.03–48.91			
Age (years)	≤55	141	44.91	39.35–50.47	0.718		
	>55	68	46.42	38.34–54.49			
Family history	No	176	43.70	38.72–48.68	0.133		
	Yes	33	54.63	43.41–65.85			
HBsAg	Negative	39	44.44	34.13–54.74	0.866		
	Positive	170	45.63	40.52–50.74			
Median size (range, cm)	≤5	73	67.38	61.44–73.31	<0.001	2.160 (1.308–3.568)	0.003
	>5	136	33.61	28.30–38.91			
Cirrhosis	No	20	42.25	27.93–56.58	0.698		
	Yes	189	45.75	40.91–50.58			
Tumor number	Single	141	51.38	45.88–56.88	<0.001	1.151 (0.713–1.857)	0.565
	Multiple	68	33.06	25.61–40.50			
Wine-drinking	No	103	49.55	42.73–56.37	0.067		
	Yes	106	41.52	35.47–47.57			
TNM stage	I–II	91	61.48	55.54–67.43	<0.001	1.980 (1.198–3.272)	0.008
	III–IV	118	33.01	27.24–38.79			
Metastasis	No	178	46.59	41.59–51.59	0.204		
	Yes	31	38.77	27.63–49.90			
BCLC stage	0-A	96	60.16	54.05–66.27	<0.001	1.159 (0.639–2.103)	0.627
	B–C	113	32.87	27.11–38.63			
AFP (ng/ml)	≤100	84	49.32	42.20–56.44	0.178		
	>100	125	42.79	36.85–48.73			
AST (U/L)	≤40	97	58.27	51.78–64.77	<0.001	1.637 (1.052–2.546)	0.029
	>40	112	34.29	28.61–39.97			
Recurrence	No	135	35.29	29.75–40.84	<0.001	2.736 (1.756–4.264)	<0.001
	Yes	74	63.93	57.79–70.67			

).

Multivariate analyses indicated that high tumor expression of BARD1 (hazard ratio [HR], 1.769; 95% CI, 1.184–2.644; *P* = 0.005), size of tumor >5 cm (HR, 2.420; 95% CI, 1.468–3.991); *P* = 0.001), TNM stage III–IV (HR, 1.698; 95% CI, 1.026–2.809; *P* = 0.039), and increased serum AST levels (HR, 1.664; 95% CI, 1.072–2.582; *P* = 0.023) were independent predictors of a shorter PFS in HCC patients (Table 2). High tumor expression of BARD1 (HR, 1.655; 95% CI, 1.100–2.489; *P* = 0.016), size of tumor >5 cm (HR, 2.160; 95% CI, 1.308–3.568); *P* = 0.003), TNM stage III-IV (HR, 1.980; 95% CI, 1.198–3.272; *P* = 0.008), and increased serum AST levels (HR, 1.637; 95% CI, 1.052–2.546; *P* = 0.029), recurrence (HR, 2.736; 95% CI, 1.756–4.264; *P* < 0.001) were significant independent prognostic factors for shorter OS in HCC patients (Table 3).

### Knockdown of BARD1 inhibited the proliferation, invasion, and migration of HCC cells

**Figure 3:**
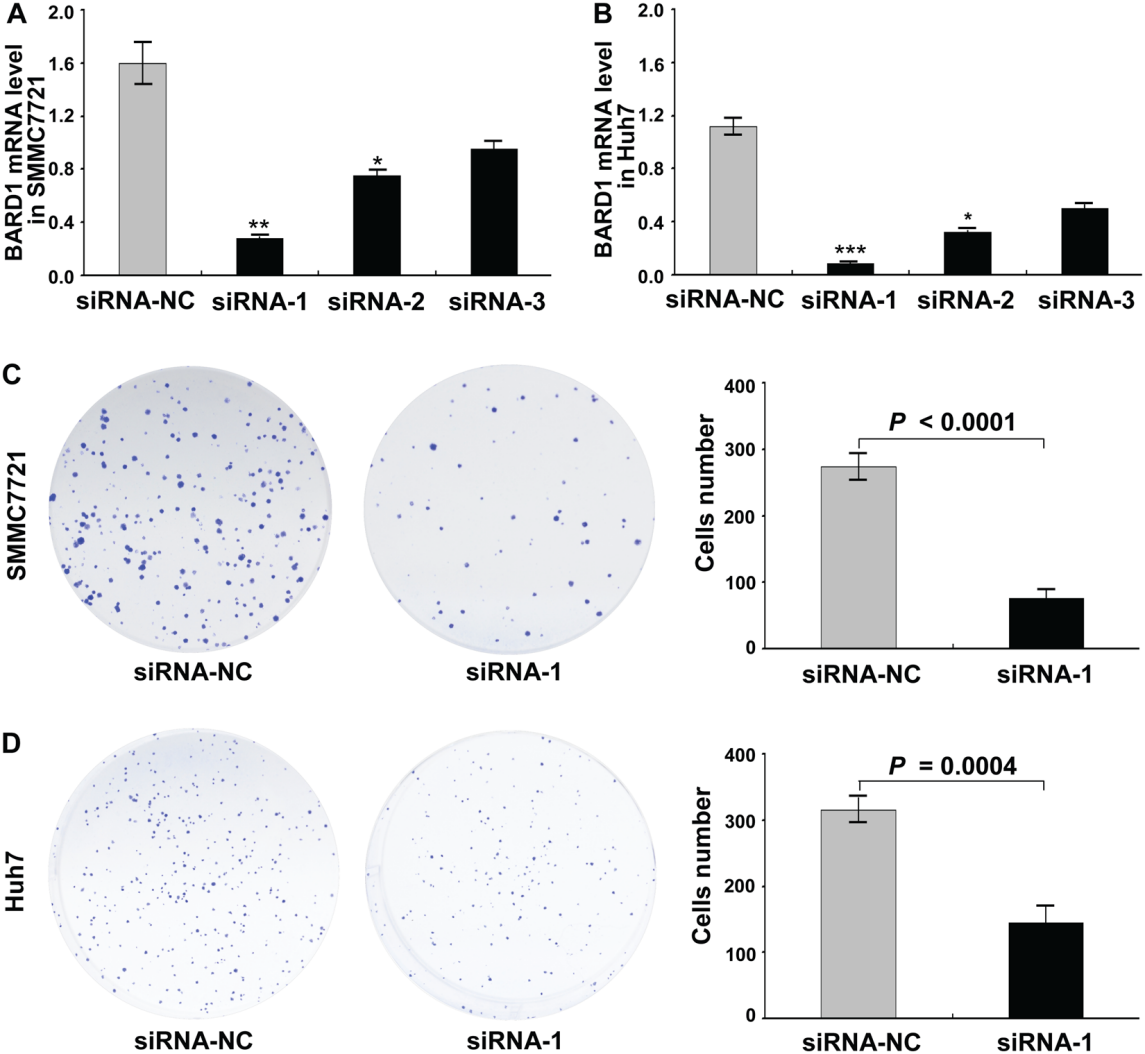
Knockdown of BARD1 inhibited the cell proliferation of HCC. (**A,B**) RNAi efficiency was demonstrated by qRT-PCR in SMMC7721 (**A**) and Huh7 (**B**) cells (*P < 0.05; **P < 0.01; ***P < 0.001). (**C**,**D**) Knockdown of BARD1 with siRNA-1 dramatically decreased cell proliferation in both SMMC7721 (**C**) and Huh7 (**D**) cells using colony formation assay, with siRNA-NC serving as a control. The histograms represent the numbers of colonies from triplicate tests (mean ±SD).

**Figure 4:**
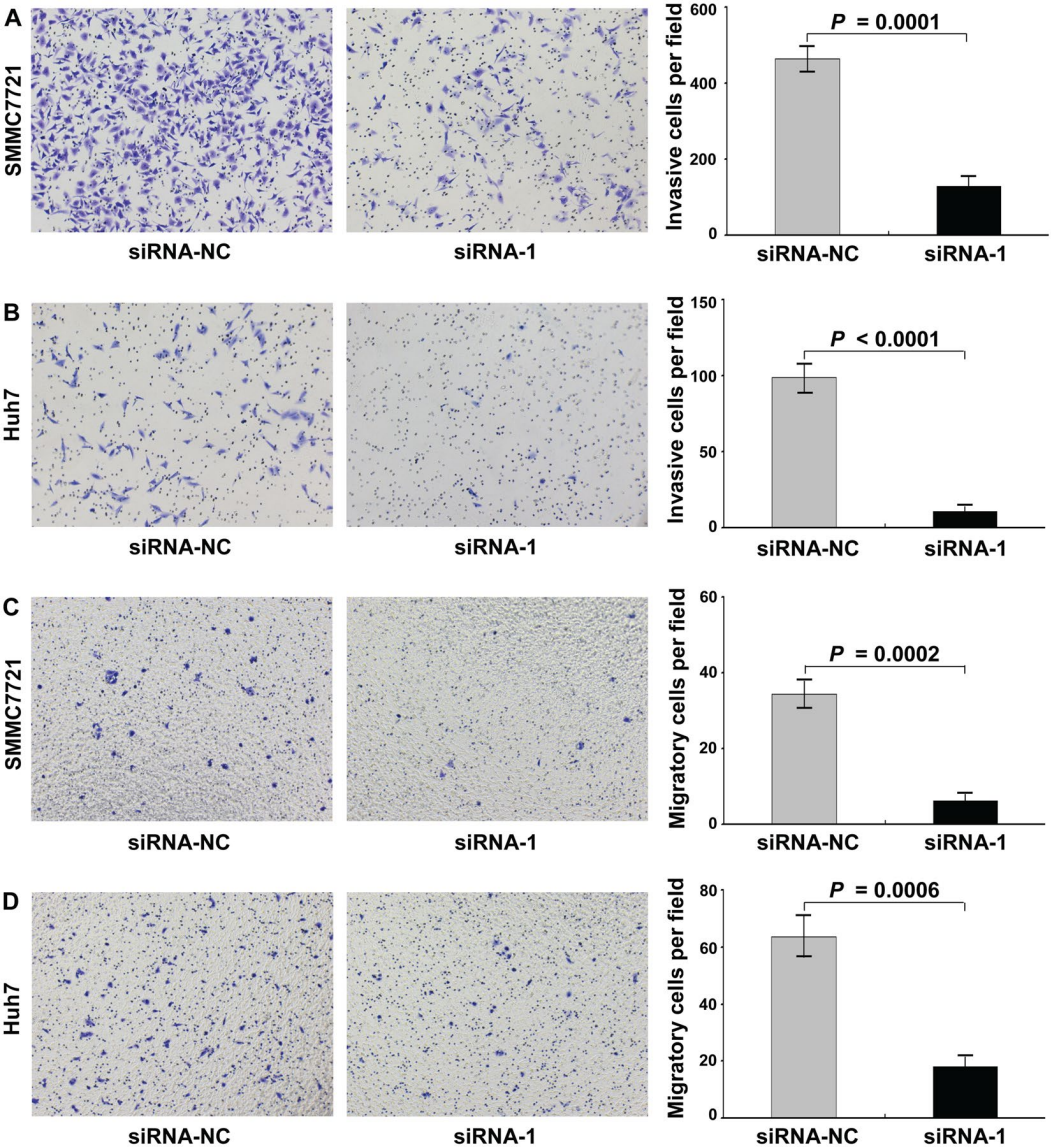
The effect of BARD 1 knockdown on the invasion and migration of HCC cells. (**A,B**) Knockdown of BARD1 with siRNA-1 dramatically decreased the invasion ability of SMMC7721 (**A**) and Huh7 (**B**) cells using Matrigel assay. (**C**,**D**) The results of *in vitro* migration assay showed that the migration ability of both SMMC7721 (C) and Huh7 (**D**) cells transfected with siRNA-1 to knock down BARD1 expression was dramatically inhibited. The numbers of invasive and migrated cells represent mean values per field (from at least 5 fields) from 3 independent experiments (right panel) (mean ± SD).

To evaluate the role of BARD1 in HCC, BARD1 was knocked down by three specific siRNAs (siRNA-1, siRNA-2, and siRNA-3) in SMMC7721 and Huh7 cells (Fig. 3A and B). As expected, knockdown of BARD1 markedly inhibited cell proliferation in both cell lines compared to the control group through colony formation assay (*P* < 0.0001 and *P* = 0.0004, respectively; Fig. 3C and D). In addition, the effects of BARD1 on malignancy of HCC cells were further evaluated by cell invasion and migration assays. The results revealed that downregulation of BARD1 dramatically reduced the invasive ability of both SMMC7721 (*P* = 0.0001, Fig. 4A) and Huh7 cells (*P* < 0.0001, Fig. 4B). Similarly, the migration assay illustrated that knockdown of BARD1 significantly decreased the migration of SMMC7721 (*P* = 0.0002, Fig. 4C) and Huh7 cells (*P* = 0.0006, Fig. 4D) compared to control group. Thus, these findings indicate that up-regulation of BARD1 may contribute to HCC progression by promoting cell proliferation, invasion and migration.

### Knockdown of BARD1 inhibited the Akt signaling pathway

**Figure 5:**
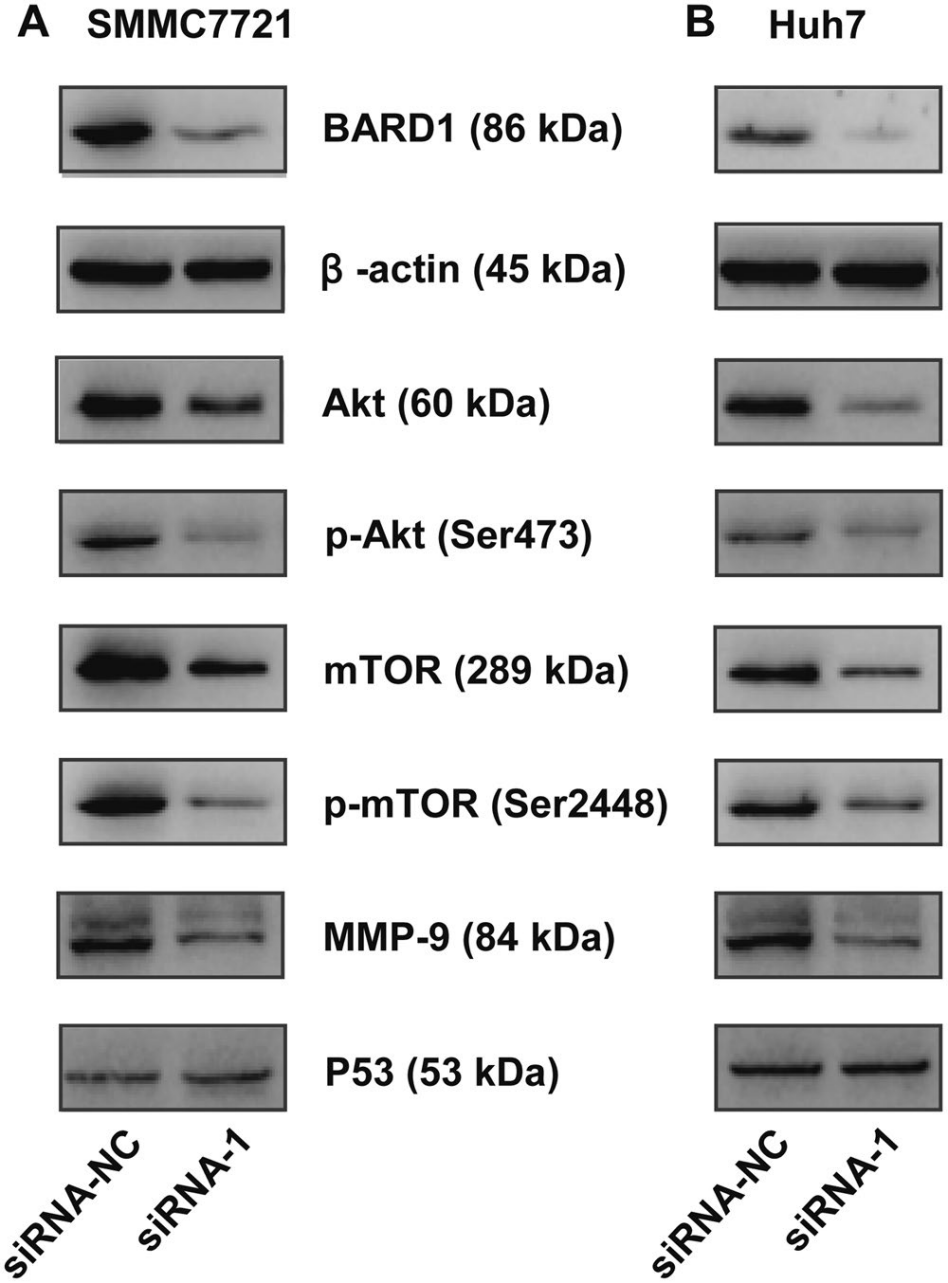
BARD1 downregulation inhibits the Akt relative signaling pathway. (**A**,**B**) Downregulation of BARD1 decreased the levels of Akt, mTOR, and MMP-9 and inhibited the phosphorylation of Akt (Ser473) and mTOR (Ser2248) in both SMMC7721 (**A**) and Huh7 (**B**) cells, except for the P53. The expressions of indicated proteins were assessed by western blot analysis with the corresponding antibodies. β-actin served as a loading control. Cropped blots are displayed for clarity. Full-size blots are presented in Supplementary Figure S1.

To explore the underlying mechanism by which BARD1 regulates the proliferation, invasion and migration of HCC cells, we further assessed its influence on the activity of Akt signaling pathway, which is well documented to be associated with tumor cell proliferation, invasion and migration. The results showed that knockdown of BARD1 evidently decreased the levels of Akt and p-Akt (Ser473) in SMMC7721 and Huh7 cells (Fig. 5A and B). Meanwhile, Akt signaling downstream target genes including mTOR, p-mTOR (Ser2448), and MMP-9 were also assessed. As expected, the levels of mTOR, p-mTOR (Ser2448), and MMP-9 were obviously reduced after knockdown of BARD1 in both cell lines (Fig. 5A and B). However, knockdown of BARD1 expression did not decrease the level of P53 (Fig. 5A and B). In conclusion, these results suggest that BARD1 promotes HCC progression through Akt signaling pathway independent of P53.

## Discussion

The high frequency of HCC metastases and disease recurrence are barriers to improving the long-term survival of HCC patients treated with hepatic resection^24, 25^. Recent studies have determined that many aberrantly expressed genes contribute to chromosomal alterations and genomic instability, which can lead to alterations in multiple cellular pathways, including cellular proliferation, survival, differentiation, and apoptosis, and may contribute to the progression of a variety of diseases, including HCC^26–28^. However, the clinical value of these mutated cancer-related genes in predicting tumor recurrence and serving as promising targets is still very limited. Thus, there is an imminent need to identify novel biomarkers to improve the treatment quality and efficiency of HCC.

BARD1 forms a stable heterodimer with BRCA1 and thereby influences a number of cellular processes that maintain genomic stability. First, the BRCA1/BARD1 heterodimer effectively promotes the homology-directed repair (HDR) of DNA double strand break (DSB) to improve chromosome stability^29^. Second, the BRCA1/BARD1 heterodimer function as a potent E3 ubiquitin ligase that stabilizes key proteins and prevents unscheduled entry into mitosis following DNA damage^30^. In addition, BARD1 forms an active dimer with BRCA1 at the centrosome and ubiquitinates γ-tubulin, which may regulate centrosome duplication^31, 32^. BARD1 gene mutations affect the function of the BRCA1/BARD1 heterodimer, which may affect cancer progression, particularly in breast and ovarian cancer. However, the functional role of BARD1 expression and the underlying significance in HCC are poorly investigated.

We found a positive correlation between BARD1 overexpression and clinical characteristics, including tumors more than 5 cm in diameter, higher AST levels, Hepatitis B virus (HBV) infection, advanced TNM stage, and later BCLC stage, which are closely related to a bad outcome of HCC. The current findings suggested that the elevation of BARD1 level may contribute to the liver injury, further hepatocarcinogenesis and development of HCC patients.

HBV is a hepadnavirus that transfers its genomic DNA to the nuclei of hepatocytes, which is then copied and replicated from generation to generation. HBV infection is a major risk factor for HCC^33, 34^. An in-depth analysis revealed that HBV and its encoded proteins may contribute to the tumorigenesis of HCC by affecting the expression of a series of cancer-related genes^35^. In our investigation, 81.34% of the HCC patients were positive for hepatitis B antigen. Interestingly, correlation analysis showed that the percentage of HCC patients who were HBV positive and exhibited elevated BARD1 expression was 60.29%, suggesting a close association between serum HBV status and the up-regulation of BARD1 in HCC. We therefore propose that BARD1 plays a crucial role in the carcinogenesis of HBV-related HCC and may serve as a novel molecular therapeutic target for further clinical treatment.

Interestingly, the overexpression of BARD1 positively correlated with AFP (>100 ng/mL) (*P* < 0.05). The serum tumor biomarker AFP is an attractive tool for routine surveillance and noninvasive diagnosis of HCC in clinical practice^36^. However, the clinical value of AFP has been challenged because of its low sensitivity and specificity. A large proportion of patients with early-stage HCC or other malignant tumors may also show an elevated serum AFP level^37^. In our study, we demonstrated that the BARD1 levels are significantly higher in HCC patients and possess a diagnostic power comparable to that of serum AFP in differentiating HCC patients from healthy individuals. Furthermore, the combination of BARD1 gene and serum AFP significantly improved early diagnosis sensibility of HCC, which is a crucial step for prolonging the survival rate of HCC patients.

These data suggest that BARD1 has the ability to promote the proliferation and growth of HCC cell lines. Matured BARD1 gene significantly impacted the sub-cellular localization and retention of BRCA1, which led to the formation of a BRCA1/BARD1 heterodimer that was not effective in regulating the HDR of DSB. These circumstances contributed to genomic instability, which propagated the uncontrolled proliferation and growth of malignant cells and accelerated the process of HCC. In addition, Cox proportional-hazards regression revealed that tumor size and clinical TNM stage possess independent prognostic significance for both PFS and OS of HCC patients, which was in accordance with previously reported findings^38^.

We found that HCC patients with low BARD1 expression levels had a higher survival rate than those with elevated BARD1 expression, and demonstrated that BARD1 was an independent prognostic factor for both PFS and OS in HCC. Accumulating evidence showed the tumor-suppressor function of BARD1^19^, however, we reported the up-regulated expression of BARD1 significantly correlated with an unfavourable prognosis in HCC, which most probably because the aberrant form of BARD1 caused by differential splicing resulted in a compromised tumor-suppressor activity with the tumor microenvironment, contributed to the tumorigenesis of HCC. This result lied in with past researches that mutated expression of BARD1 is closely connected with poor prognosis in breast and ovarian cancer^39^. We therefore concluded the mutations and epigenetic modifications in BARD1 gene may lead to the loss of tumor-suppressor function and further favor the HCC carcinogenesis.

The *in vitro* experiments demonstrated that knockdown of BARD1 significantly inhibited the proliferation, invasion and migration of HCC cells. In order to reveal the underlying mechanisms, several important molecules which are known to result in the promotion of tumor progression were performed by western blot analysis. Our data showed that knockdown of BARD1 could downregulate the levels of Akt, mTOR and MMP-9, as well as their phosphorylation levels. These results suggest that BARD1 may promote HCC progression via targeting AKT signaling pathway, and the up-regulation mechanism in HCC should not be control by P53, instead, it may be regulated by the other mechanisms.

In summary, we showed that BARD1 was significantly up-regulated in HCC tumor tissues and that BARD1 up-regulation was significantly associated with a lower HCC patient survival rate and advanced stage. Up-regulation of BARD1 may contribute to HCC carcinogenesis by promoting cell proliferation, invasion and migration through targeting Akt signaling. Our data indicate the need for further investigation, preferably in large population studies, before BARD1 can be used as a novel biomarker or molecular target for the effective treatment of HCC patients in routine clinical practice.

## Electronic supplementary material

Supplementary Figure S1
