# Supplementary material for: Up-regulation of BRCA1-associated RING Domain 1 Promotes Hepatocellular Carcinoma Progression by Targeting Akt Signaling
Source: Sci Rep. 2017 Aug 9;7:7649. doi: 10.1038/s41598-017-07962-7 (PMC5550490; doi:10.1038/s41598-017-07962-7)
Supplement: Supplementary file 1 — Supplementary Figure S1 [file 41598_2017_7962_MOESM1_ESM.doc]

**Supplementary Information**

**Up-regulation of BRCA1-associated RING Domain 1 Promotes Hepatocellular Carcinoma Progression by Targeting** **Akt Signaling**

Yan Liao^1, 2*^, Shengguang Yuan^3*^, Xinhuang Chen^1*^, Pengpeng Zhu^1^, Jun Li^1^, Liling Qin^1^, Weijia Liao^1^

^1^ Laboratory of Hepatobiliary and Pancreatic Surgery, Affiliated Hospital of Guilin Medical University, Guilin, Guangxi, P.R. China

^2^ Disease Prevention and Control Center of Guilin, Guilin, Guangxi, P.R. China

^3^ Department of Hepatobiliary and Pancreatic Surgery, Affiliated Hospital of Guilin Medical University, Guilin, Guangxi, P.R. China

*These authors contributed equally to this work.

Correspondence and requests for materials should be addressed to W.-J.L. (email: liaoweijia288@163.com)


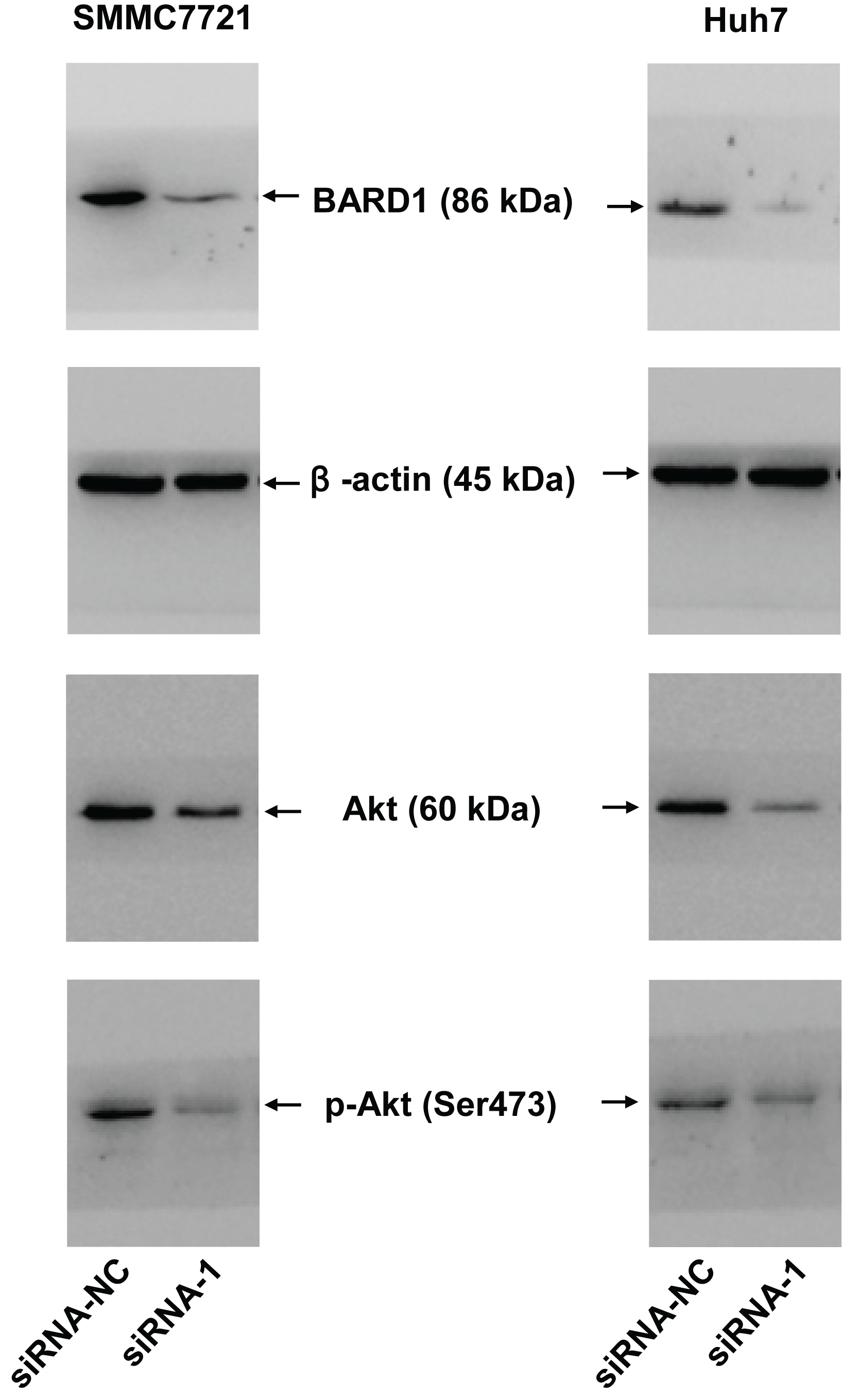

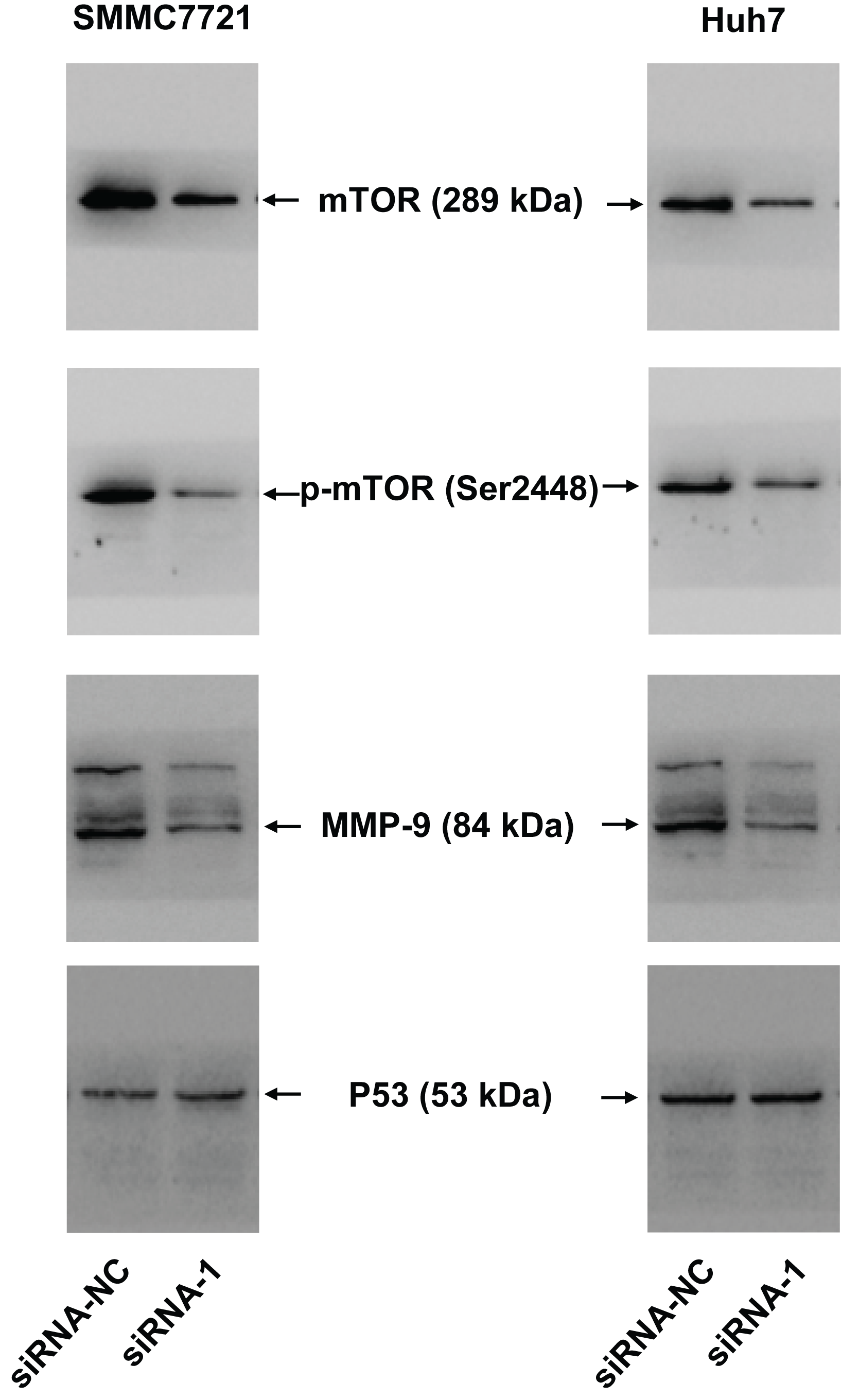


**Supplementary Figure S1. Full-size blots of Figure 5.**

Downregulation of BARD1 decreased the levels of Akt, mTOR, and MMP-9 and inhibited the phosphorylation of Akt (Ser473) and mTOR (Ser2248) in both SMMC7721 and Huh7 cells, except for the P53. The expressions of indicated proteins were assessed by western blot analysis with the corresponding antibodies. β-actin served as a loading control.
